# Folate Catabolites in Spot Urine as Non-Invasive Biomarkers of Folate Status during Habitual Intake and Folic Acid Supplementation

**DOI:** 10.1371/journal.pone.0056194

**Published:** 2013-02-14

**Authors:** Mareile Niesser, Hans Demmelmair, Thea Weith, Diego Moretti, Astrid Rauh-Pfeiffer, Marola van Lipzig, Wouter Vaes, Berthold Koletzko, Wolfgang Peissner

**Affiliations:** 1 Division of Metabolic and Nutritional Medicine, Dr. von Hauner Children’s Hospital Medical Center of Ludwig Maximilians University, Munich, Germany; 2 Unilever Food and Health Research Institute, Unilever Research & Development, Vlaardingen, The Netherlands; 3 Laboratory of Human Nutrition, Swiss Federal Institute of Technology. Zürich, Switzerland; 4 TNO Innovation for Life, Zeist, The Netherlands; Aga Khan University, Pakistan

## Abstract

**Background:**

Folate status, as reflected by red blood cell (RCF) and plasma folates (PF), is related to health and disease risk. Folate degradation products para-aminobenzoylglutamate (pABG) and para-acetamidobenzoylglutamate (apABG) in 24 hour urine have recently been shown to correlate with blood folate.

**Aim:**

Since blood sampling and collection of 24 hour urine are cumbersome, we investigated whether the determination of urinary folate catabolites in fasted spot urine is a suitable non-invasive biomarker for folate status in subjects before and during folic acid supplementation.

**Study Design and Methods:**

Immediate effects of oral folic acid bolus intake on urinary folate catabolites were assessed in a short-term pre-study. In the main study we included 53 healthy men. Of these, 29 were selected for a 12 week folic acid supplementation (400 µg). Blood, 24 hour and spot urine were collected at baseline and after 6 and 12 weeks and PF, RCF, urinary apABG and pABG were determined.

**Results:**

Intake of a 400 µg folic acid bolus resulted in immediate increase of urinary catabolites. In the main study pABG and apABG concentrations in spot urine correlated well with their excretion in 24 hour urine. In healthy men consuming habitual diet, pABG showed closer correlation with PF (r_s_ = 0.676) and RCF (r_s_ = 0.649) than apABG (r_s_ = 0.264, ns and 0.543). Supplementation led to significantly increased folate in plasma and red cells as well as elevated urinary folate catabolites, while only pABG correlated significantly with PF (r_s_ = 0.574) after 12 weeks.

**Conclusion:**

Quantification of folate catabolites in fasted spot urine seems suitable as a non-invasive alternative to blood or 24 hour urine analysis for evaluation of folate status in populations consuming habitual diet. In non-steady-state conditions (folic acid supplementation) correlations between folate marker (RCF, PF, urinary catabolites) decrease due to differing kinetics.

## Introduction

Suboptimal folate status is related to higher risks of neural tube defects [1], [2], decline of cognitive performance in dementia and Alzheimer’s disease [3], depression [4], heart diseases, increased homocysteine levels [5], [6], [7] and cancer risks [8], [9]. Recently higher folate status has been associated with growth and progression of preexisting cancerous lesions [10], [11], [12], [13], [14], which raises concerns in respect to excessively high folate intakes and underscores the relevance of the determination of folate status for the identification of increased disease risks.

Red blood cell folate (RCF) and plasma folate (PF) levels are widely accepted direct biochemical indicators of folate status, but determination requires blood sampling. RCF is considered as a long term marker as the red blood cell folate pool turns over slowly [2], [15]. It represents folate stores [2], [15] and is widely used as a marker of folate status. In contrast, circulating PF changes rapidly after folate intake [16].

Gregory et al. [17] and McPartlin et al. [18] demonstrated that folates are catabolized in the liver to para-aminobenzoylglutamate (pABG), which is excreted in urine predominantly after acetylation to para-acetamidobenzoylglutamate (apABG). Folate catabolite excretion in urine decreases with low dietary folate intake and increases after folate supplementation [19]. Thus, similar to RCF and PF, the urinary folate catabolites pABG and apABG might reflect folate status.

Only limited data are currently available comparing blood folate contents with urinary folate catabolite excretion. Kim et al. [20] analyzed folate catabolite excretion, blood folate status markers and dietary folate intake and found significant correlations between these markers. Wolfe et al. [19] determined blood and urine markers in postmenopausal women on controlled diets with deficient and adequate folate content and found a significant correlation between the sum of pABG and apABG excretion in urine and PF. While these findings suggest a close relationship between blood folate and urinary catabolite excretion during low folate intake, the correlation has not been fully investigated during folic acid supplementation.

Wolfe et al. and Kim et al. [19], [20] studied relations between folate catabolite excretion in 24 hour urine and blood folates.

Collection of blood samples as well as 24 hour urine are laborious, costly and invasive and 24 hour urine collection may be impaired by loss of volume or degradation of analytes over time [21], [22]. Therefore we studied the relation between urinary folate catabolite concentrations relative to creatinine in spot urine samples and folate levels in plasma and red blood cells. Although diurnal variations are averaged out by 24 hour collection, there are still indications that daily excretion of urinary pABG and apABG may not only be determined by long-term folate storage but also influenced by short-term folic acid intake [23]. Thus, we evaluated the relationship between urinary folate catabolites and blood folate markers in healthy males before (baseline) and during a 12 week period of folic acid supplement intake. Prior to this, short-term effects of a folic acid bolus on urinary catabolite concentrations were studied to evaluate limitations that have to be observed for collection of eligible spot urine. The calculated ratio between folate catabolites pABG and apABG (ratio_p/ap_) was furthermore used to detect potentially biased spot urine.

## Subjects and Methods

### Study Participants

**Figure 1:**
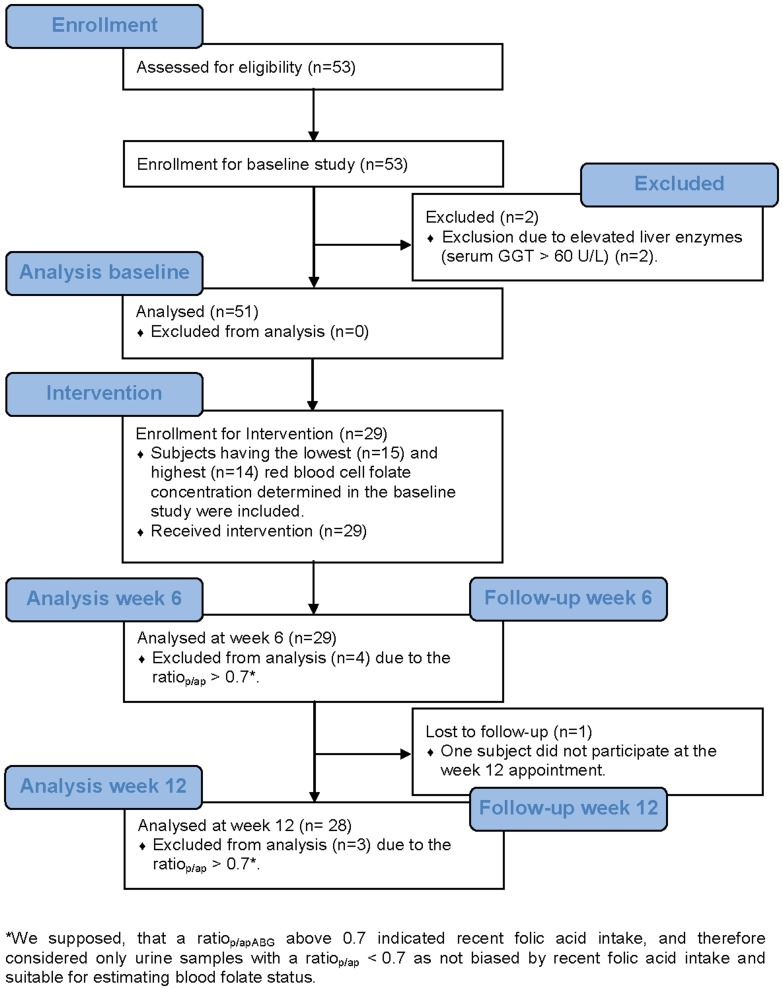
Flow chart of study selection process.

The sample size estimation was performed for the baseline study. We aimed to detect a correlation of at least 0.35 between urinary folate catabolites and blood folate as corresponding correlations had been observed between various measures of dietary folate intake and blood folate markers [24], [25]. To detect such a correlation as statistically significant with a power 80% and α of 0.05, samples from 49 subjects have to be analyzed [26]. Thus, 53 apparently healthy men were enrolled into the baseline study to compensate for eventually later detected exclusion criteria (Figure 1). The subsequent 12 week intervention of 29 subjects with an intake of 400 µg folic acid per day was accomplished based on an explorative design without explicit power calculation.

The objectives and procedures of the study were explained and all participants provided written informed consent. The research protocol was approved by the Ethical Committee of the Medical Faculty of the University of Munich and the study was registered at clinicaltrials.gov under NCT00689949. Requirements for inclusion into the study were no reported diagnosis of intestinal, renal or thyroid disease, normal full blood count, no alcohol or drug abuse (max 1 alcoholic drink per day), and no concurrent participation in other clinical studies. Medication during the last three month, which was assumed to interfere with folate status, was an exclusion criterion. Test persons having a regular consumption of vitamin supplements (including folate) were not excluded, since we aimed to cover a wide range of folate levels.

### Study Design and Sample Collection

Data were collected between May and October 2008. The baseline study included two examinations, 21 days apart. Each examination was arranged in the morning after ≥12 hours of fasting. After discarding the first morning void urine at home, the second fasted spontaneous urine was collected in urine containers (70 mL, Sarstedt, Germany) at the study center (Division of Metabolic and Nutritional Medicine, Dr. von Hauner Children’s Hospital, Medical Center of Ludwig Maximilians University, Munich, Germany). Directly after collection urines were centrifuged (2000×g, 5 min) or filtered (syringe filter 25 mm, 0.2 µm nylon membrane, VWR, Germany), then aliquoted and stored at −80°C. Previous experiments in our laboratory had shown that folate catabolites were stable in urine for more than five days at room temperature. (Niesser et al., submitted) At the same time blood samples were collected in tubes containing EDTA and heparin (EDTA-, Heparin Monovette, Sarstedt, Germany). For determination of whole blood folate, 100 µL of heparinized, whole blood was mixed with 900 µL fresh ascorbic acid solution (1%) for hemolyzation in the dark (30 min) and stored at −80°C. After centrifugation of Heparin-Monovettes (1700×g, 5 min), the plasma was aliquoted into Eppendorf tubes and stored at −80°C until analysis of PF.

24 hour urine was collected during the day and night preceding the second examination in a light protecting urine bottle (Urisafe, 3.0 L PP, VWR, Germany) prefilled with 30 mL hydrochloric acid (1N) for limitation of microbial growth and preservation of sample quality. Total urine volume was recorded for each participant. After centrifugation or filtration, urine aliquots were stored at −80°C until analysis.

From the participants of the baseline study the fifteen participants having the lowest RCF concentration and the fourteen subjects having the highest RCF concentration, determined in the baseline study, were included in the subsequent intervention with folic acid (intervention study) (Figure 1). They consumed 400 µg folic acid (Folverlan, 0.4 mg, Verla-Pharm, Germany) daily for twelve weeks. Morning spot urine and blood samples were collected after 6 and 12 weeks of supplementation (Figure 1).

For the investigation of urinary folate catabolite excretion shortly after folate ingestion, a two day short-term study was conducted, including seven apparently healthy, lean volunteers. At day one, participants delivered urine samples four times during a fasting period of three hours, starting with the second fasted urine, taken in the morning after an overnight fasting period. Urine samples two, three and four were collected one, two and three hours after the first urine collection. Experimental conditions on day two were identical to day one, but after collection of the first urine sample a 400 µg folic acid tablet (Folverlan, 0.4 mg, Verla-Pharm, Germany) was ingested.

### Urine Analysis

Urine folate catabolites pABG and apABG were quantified by high performance liquid chromatography combined with tandem mass-spectrometry (LC-MS/MS) (Niesser et al., submitted). Briefly, 80 µL urine were transferred into a well plate which was pre-filled with acetonitrile, methanol and hydrochloric acid containing (^13^C_2_D_3_)apABG (synthesized according to Sokoro et al. [27]) and (^13^C_5_)pABG (synthesized according to Niesser et al., submitted) as internal standards. The well plate was shaken and cooled for protein precipitation before centrifugation. The supernatants were transferred into a second well plate and dried under nitrogen. For derivatization samples were re-dissolved in 3N-butanolic hydrogen chloride and incubated at 60°C for 10 min. Derivatization was stopped by cooling on crushed ice, butanolic hydrogen chloride was evaporated under nitrogen and after taking up the derivates into methanol, water and formic acid (50∶50∶0.1), samples were analyzed by LC-MS/MS (Agilent Technologies 1200SL series HPLC system, Germany; API 4000 QTrap, AB Sciex, Germany). Chromatography was performed on an Agilent Zorbax SB C18 column (2.1×50 mm, particle size 1.8 µm, Agilent, Germany). Atmospheric pressure chemical ionization with mass transitions 379 → 120, 381 → 120, 384 → 120, 421 → 162, 426 → 165, were used for detection of pABG, (^13^C_2_)pABG, (^13^C_5_)pABG, ***apABG and*** (^13^C_2_D_3_)apABG, respectively. Relative concentrations of urinary folate catabolites, determined in a calibration range of 2 to 1000 nmol/L, were calculated as nmol per mmol creatinine.

Creatinine was determined by a routine assay using the Creatinine Jaffé method [28] on a Cobas (Roche Diagnostics, Germany). Urinary methylmalonic acid (MMA) was analyzed by isotope dilution GC-MS as described by Matchar et al. [29]. Urinary MMA concentrations were normalized against creatinine concentrations. Creatinine and MMA were analyzed at the Institute for Clinical Chemistry, University of Munich Medical Center.

### Blood Analysis

Whole blood folate, PF and urinary folate were assessed at TNO Innovation for Life, Zeist by a microbiological assay according to O’Broin et al. [30] using a chloramphenicol resistant, cyro-conserved bacterial strain of L. casei and photometric determination of bacterial growth. The lowest calibrator concentration was considered as the lower limit of quantification of folate in the assay (PF: 2.3 nmol/L; urinary folate: 2.9 nmol/L). RCF was calculated from whole-blood and plasma folate concentrations and hematocrit. Analyses described below were performed at the Institute for Clinical Chemistry, University of Munich, Medical Center. Determination of hematocrit was done using a Sysmex XT 1800i fluorescence flow cytometer (Sysmex, Germany). Concentrations of homocysteine in EDTA-plasma and Vitamin B_12_ in serum were determined using a fully automated competitive chemiluminescence immunoassay on an AVIDA Centaur XP immunoassay System (Siemens Healthcare Diagnostics, Germany). Vitamin B_6_ was analyzed as pyridoxal-5′-phosphate from EDTA-blood using a chromatographic method (Vitamin B_6_ reagent kit for HPLC analysis, Chromsystems, Germany).

### Criteria for Exclusion of Baseline and Intervention Study Data

Two of the initially recruited 53 participants were excluded from statistical analysis because of elevated liver enzymes (serum gamma-glutamyl transpeptidase (GGT) >60 U/L [31]). For technical reasons RCF concentrations from six participants at the second baseline examination were not available and concentrations from the first examination were used instead of means. Only the first examination was included for two participants, as a ratio_p/ap_>0.7 was found at the second examination indicating potential bias of urinary folate catabolite concentrations by folate intake shortly before sampling (see paragraph on short-term study). Accordingly, also the 24 hour urines of these two participants were excluded. Data of four participants at week 6 and three at week 12 of the intervention were excluded from statistical analysis for the same reason. One subject did not participate at the week 12 examination. Finally, 51 subjects were statistically analyzed at baseline and 25 at weeks 6 and 12 (Figure 1).

### Statistical Analysis

Means per participant were calculated as baseline concentration for all determined blood and urine analytes from examination one and two if valid results were available for both time points. Quantified analytes of the baseline and intervention study (week 6 and 12) are given as medians with inter quartile range (IQR) per analyte.

Blood folate concentration RCF and PF were used as the reference values. For evaluation of folate catabolites concentrations in 24 hour urine were correlated with those in spot urine and both with blood folate concentrations at baseline and during supplementation in the intervention study, respectively. As data was not normally distributed, Spearman’s non-parametric rank correlation was used to analyze relations of biochemical parameters within the baseline and intervention study. (Spearman’s rank correlation coefficient hereinafter is abbreviated as r_s_.) Wilcoxon’s rank test was applied for evaluating changes by supplementation in the short-term study and in the intervention study. Statistical analyses were performed with Statistica 10 (StatSoft GmbH, Germany) and PASW Statistics 18 (IBM, USA).

## Results and Discussion

### Increase of Urinary Folate Catabolites after Folic Acid Intake in the Short-term Study

**Figure 2:**
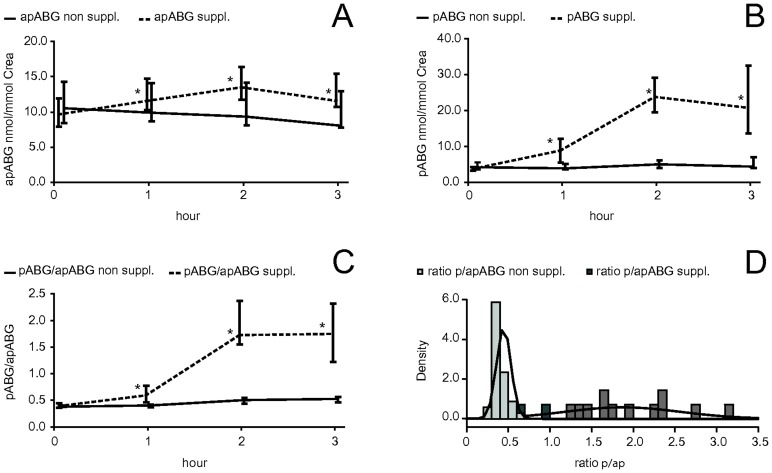
Time course of urinary apABG and pABG after folic acid intake (short-term study). Time course (medians, error bars = inter quartile range (IQR)) of urinary apABG (nmol/mmol creatinine) (A) and pABG (nmol/mmol creatinine) (B), in fasted state (black line) and after oral intake of 400 µg folic acid (dashed line) during the first three hours. (C) Time course of the ratio between pABG and apABG (ratio_p/ap_) in spot urine without (black) and with (dashed line) intake of folic acid at time 0. Significant differences from time point 0 are marked with an asterisk (*: p≤0.05, Wilcoxon’s test). (D) Histograms of observed frequencies of ratio_p/ap_ in spot urine in non-dosed state (light grey bars) and 2 h after oral folate intake (dark grey bars), displayed with continuous kernel density function estimates (black lines).

Kinetic modeling of stable isotope tracer data has shown that most of the body folate is incorporated into pools with slow turnover, e.g. half live of around 90 days [32], [33]. If urinary folate catabolites were solely derived from slow body folate pools [19], [34], [35], only slow changes of catabolite concentrations in urine without immediate dietary effects would be expected. However, in the short-term study we found urinary apABG and pABG to increase significantly (p≤0.05) after oral ingestion of 400 µg folic acid (Figure 2A, 2B dashed line). This increase of urinary apABG and pABG excretion was not detectable in the group not receiving folic acid supplements (Figure 2A, 2B black line). A portion of ingested folic acid might have been catabolized shortly after uptake, resulting in an increase of urinary apABG and especially pABG.

Scott et al. [36] investigated the distribution of radiolabeled folate in rats and found different distribution patterns among tissues. There were fast and slow turnover pools within the body [36], thus urinary excretion may be determined by rapid and slow excretion rates which may explain both urinary folate catabolite increase shortly after folate intake in the short-term study and increased catabolite excretion after long-term supplementation.

The contribution of dietary pABG and of pABG formed by pre-absorptive cleavage of folate to urinary pABG and apABG excretion has been reported to be very small [37]. Rather urinary pABG may at least partly arise from nonspecific extracellular degradation of folates either in circulation or bladder as suggested by Geoghegan et al. [38]. Thus, the 300% increase of urinary pABG concentration after folic acid ingestion in the short-term study could be mainly due to fast breakdown of folic acid. It remains to be determined whether an increase of folate catabolite excretion only occurs after folic acid intake or also after the intake of polyglutamated 5-methyltetrahydofolate from food.

Urinary apABG arises from endogenous folate after breakdown and acetylation by arylamine *N*-acetyltransferase I (NAT1) in the cytosol [38], [39], which explains the relatively small increase of apABG in contrast to pABG in the short-term study. NAT1 is widely distributed in the body including the cytoplasm of epithelial cells [39]. Phenotypes with low or higher than normal acetylation activity in some tissues have been reported, which result in inter-personal variation of pABG acetylation [39] and may explain inter-individual differences of fasted apABG levels and variations of the apABG increase after the ingestion of folic acid in the short-term study.

For the calculated ratio of pABG to apABG (ratio_p/ap_) a median (IQR) value of 0.42 (0.38; 0.49) was found in non-dosed state (Figure 2C, black line) which is similar to previous reports [18], [19], [40], [41]. Shortly after oral intake of folic acid the ratio_p/ap_ increased significantly to 1.74 (1.43; 2.36) (Figure 2C, dashed line). Observed distributions of ratio_p/ap_ in dosed versus non-dosed state were compared (Figure 2D). Ratio_p/ap_ after folic acid intake (Figure 2D, dark grey bars) was clearly higher and showed a much higher variability than ratio_p/ap_ measured in non-dosed state (Figure 2D, light grey bars). The lowest observed ratio_p/ap_ in dosed state was 0.65 and the highest in non-dosed state was 0.70. Thus, immediate effects of folic acid intake increased urinary concentrations of pABG much more than that of apABG, thereby altering the ratio between both analytes. Based on this observation we suppose a ratio_p/ap_ above 0.7 indicated recent folic acid intake. We therefore considered only urine samples with a ratio_p/ap_<0.7 as not biased by recent folic acid intake and suitable for estimating blood folate status.

Since folic acid ingestion showed a clear short-term influence on apABG and particularly pABG levels, estimation of folate status from catabolite concentrations in spot urine requires overnight fasting periods of more than 12 hours before sampling to minimize interference. Although first morning voids are generally recommended [42], they may still be influenced by folates which were ingested before the fasting period, therefore we generally analyzed the second fasted morning urine, collected 1 to 2 hours after discarding the first morning void. Spot urine collected according to this protocol was most likely produced exclusively during periods where no absorption of folates from the intestinal tract was occurring, therefore mirroring folate release from body storage as closely as possible.

### Aims of Baseline and Intervention Study

The aim of our study was to evaluate the estimation of folate status by quantifying urinary pABG and apABG, as a non-invasive alternative to the analysis blood folate. Blood folate concentration RCF and PF were used as the reference values, so we compared concentrations of 24 hour urine to spot urine and both to blood folates at baseline. During supplementation blood folate concentrations were only related to catabolite concentrations in spot urine.

### Study Population of Baseline and Intervention Study

In routine analysis blood and urine parameters in the reference ranges were only found for 51 of 53 participants (data not shown), since two participants showed elevated liver enzymes (serum GGT >60 U/L [31]). Quantitative results of blood and urine analyses of the baseline study are given in Table 1. Creatinine excretion per day showed no indication of abnormal kidney function in any participant (Table 1) [22], which might have interfered with urinary folate catabolites excretion. No correlations of age, height, weight, body mass index (BMI), hematocrit or creatinine excretion per day with PF or RCF were observed (Table 1).

**Table 1:** Description of participants and biochemical parameters during habitual diet (baseline).

Collection mode	Characteristic	Median (IQR) baseline (n = 51)	Correlation with PF (nmol/L)	Correlation with RCF (nmol/L)
	Age (years)	26.5 (23.3; 30.0)	0.105	0.128
	Height (m)	1.79 (1.75; 1.83)	0.044	0.001
	Weight (kg)	74.0 (70.0; 86.5)	0.009	0.040
	BMI	23.4 (21.5; 24.9)	0.016	0.072
**Blood**	Folate, red blood cells (nmol/L)	731 (591; 860)	0.781**	1
	Folate, plasma (nmol/L)	14.3 (11.3; 21.5)	1	0.781**
	Homocysteine, plasma (µmol/L)	11.5 (10.0; 13.1)	−0.420*	−0.222
	Vitamin B_6_, EDTA blood (µg/L)	25.4 (22.5; 31.3)	0.242	0.195
	Vitamin B_12_, EDTA blood (ng/L)	439 (393; 474)	0.056	−0.091
	Hematokrit (%)	43.8 (42.0; 45.4)	−0.022	0.079
**Spot urine**	pABG (nmol/mmol creatinine)	3.59 (2.98; 4.32)	0.676**	0.649**
	apABG (nmol/mmol creatinine)	10.9 (8.86; 12.3)	0.264	0.543**
	Folate (nmol/mmol creatinine)	0.55 (0.38; 0.78)	0.540**	0.423*
	MMA (mg/g creatinine)†	1.64 (1.35; 1.99)	−0.216	−0.196
	Ratio_p/ap_	0.34 (0.29; 0.40)	0.349*	0.050
**24 hour urine**	pABG (nmol/24 hour)	71.2 (58.7; 95.2)	0.336*	0.440*
	apABG (nmol/24 hour)	211 (183; 263)	0.073	0.290
	Folate (nmol/24 hour)	7.74 (4.17; 11.5)	0.473**	0.529**
	Ratio_p/ap_	0.36 (0.30; 0.40)	0.335*	0.149
	Creatinine (mmol/24 hour)	17.4 (15.6; 19.6)	−0.087	−0.029
	Urine volume (mL)	1720 (1380; 2380)	0.058	0.042

Values are given as median, IQR in parentheses. Results of the two baseline measurements were averaged, if valid data were available from both examinations. Relations between variables were analyzed by Spearman’s rank correlation. Significance level:

* p<0.05,

** p<0.001. pABG: para-aminobenzoylglutamate, apABG: para-acetamidobenzoylglutamate, MMA: methylmalonic acid, ratio_p/ap_: ratio of pABG to apABG.

† One extreme outlier was excluded.

### Comparison of Folate Catabolites in 24 Hour and Spot Urine at Baseline

**Figure 3:**
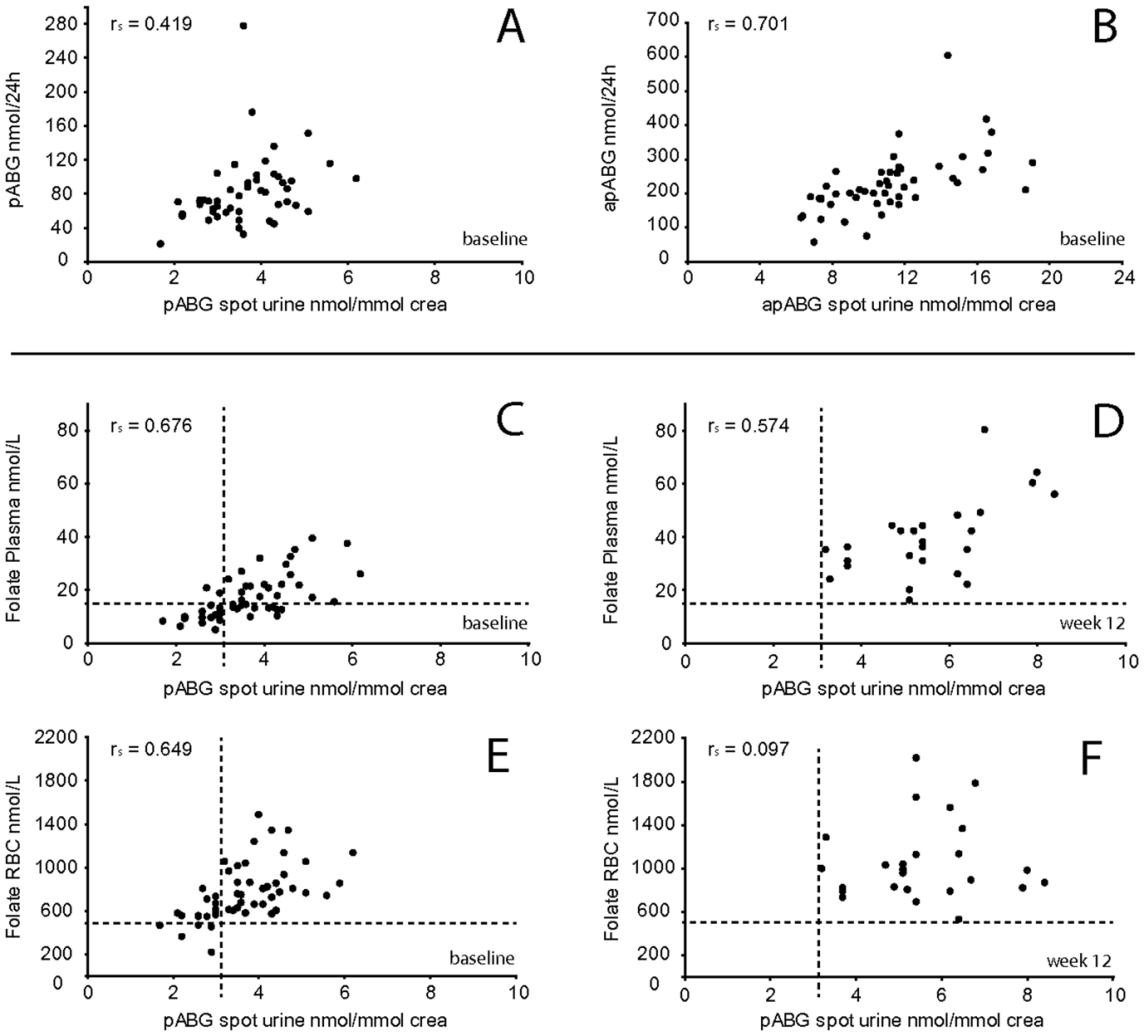
Correlation of folate catabolites in spot urine with 24 hour urine and blood folate concentrations. ***Comparison of folate catabolites in spot urine and 24 hour urine.*** Creatinine normalized concentration of para-aminobenzoylglutamate (pABG) (A) and para-acetamidobenzoylglutamate (apABG) (B) in spot urine is correlated with daily excretion in 24 hour urine. ***Correlations of urinary pABG with plasma and red blood cell folate, a comparison between baseline and week 12.*** Scatterplots for para-aminobenzoylglutamate (pABG)/creatinine in spot urine related to plasma folate and red blood cell folate concentrations, at baseline (C, E, n = 51) and week 12 (D, F, n = 25). For ease of comparison, dashed lines in C, D, E and F indicate the lowest value of the respective biomarker at week 12.

To our knowledge, no studies evaluating urinary folate catabolite concentrations in spot urine have been published so far. Excretions in 24 hour urine have been related to blood folate concentrations and correlations between folate catabolite excretion and the blood folate status have been reported (r ≈ 0.4) [19], [20]. In the present study correlations between blood folates and catabolite excretions in 24 hour urine equaled reported relations in respect to urinary pABG (PF: r_s_ = 0.336, p<0.05, RCF: r_s_ = 0.440, p<0.05, Table 1). Also urinary apABG excretion was by trend related to RCF (r_s_ = 0.290, p>0.05). Furthermore spearman’s rank correlation between folate catabolites concentration in spot urine and excretion in 24 hour urine in the baseline study was 0.419 (p<0.005) for pABG, and 0.701 (p<0.001) for apABG (Figure 3A, 3B). Since correlations between blood folates and catabolite concentrations in spot urine were stronger, than those of blood folates and catabolite excretions in 24 hour urine (Table 1), collection of 24 hour urine and determination of urinary excretions per day seemed not necessary for non-invasive evaluation of blood folate status via urinary folate catabolites.

Other authors have reported lower pABG and apABG excretion in 24 hour urine [18], [19], [20], [34], [40], [41] than the 71.2 nmol pABG and 211 nmol apABG per day we have found. This might be related to methodological differences, as analytical procedures, including de-acetylation of apABG [18], [19], [34], [41] or pre-concentration of folate catabolites without internal standard [41], are more susceptible to loss of analytes than direct analysis by LC-MS/MS. Furthermore, habitual folate intake may be higher in our study population than in previously studied subjects, e.g. in areas with less common folic acid fortification of foods [43].

Daily creatinine excretion of all participants was within previously published ranges [22] and creatinine concentrations in 24 hour urine (mmol/L) were highly negatively correlated with their appertaining urine volumes (r_s_ = −0.916, p<0.001). This indicated correct and complete urine sampling, although this has been a problem in other studies [21], [22].

### Relations between Blood Folates RCF and PF and Urinary Folate Catabolites pABG and apABG at Baseline

The baseline study showed significant correlation of RCF with PF and of both with pABG in spot urine (Figure 3C, 3E) as well as 24 hour urine (Table 1). We found a significant correlation between apABG and RCF in spot urine, but not in urine that was collected for 24 hours (Table 1). Kim et al. [20] recently published close correlations of pABG in 24 hour urine with PF, and between apABG and RCF. They concluded that pABG reflects recent folate intake, whereas apABG accounts for whole body folate. Wolfe et al. [19] correlated the sum of pABG and apABG, as ‘total pABG’, with blood folates and found a slightly higher correlation with RCF than with PF. In both studies urinary apABG was reported as the more abundant folate catabolite [19], [20]. We also found higher concentrations in spot urine and higher excretions in 24 hour urine of apABG than of pABG (Table 1), which confirmed apABG as the main urinary folate catabolite, at least during fasting. Nevertheless, RCF correlated closer with pABG than with apABG and PF only showed non-significant correlations (r_s_ <0.3, p>0.05) with apABG concentrations in spot and 24 hour urine. In contrast, pABG in spot as well as 24 hour urine correlated significantly with PF. This is in line with the assumption that fast turnover pools are well reflected by urinary pABG, while a closer relationship of slower turnover pools to apABG than to pABG could not be confirmed.

### Intervention Study: 12– Week Oral Supplementation with Folic Acid

**Figure 4:**
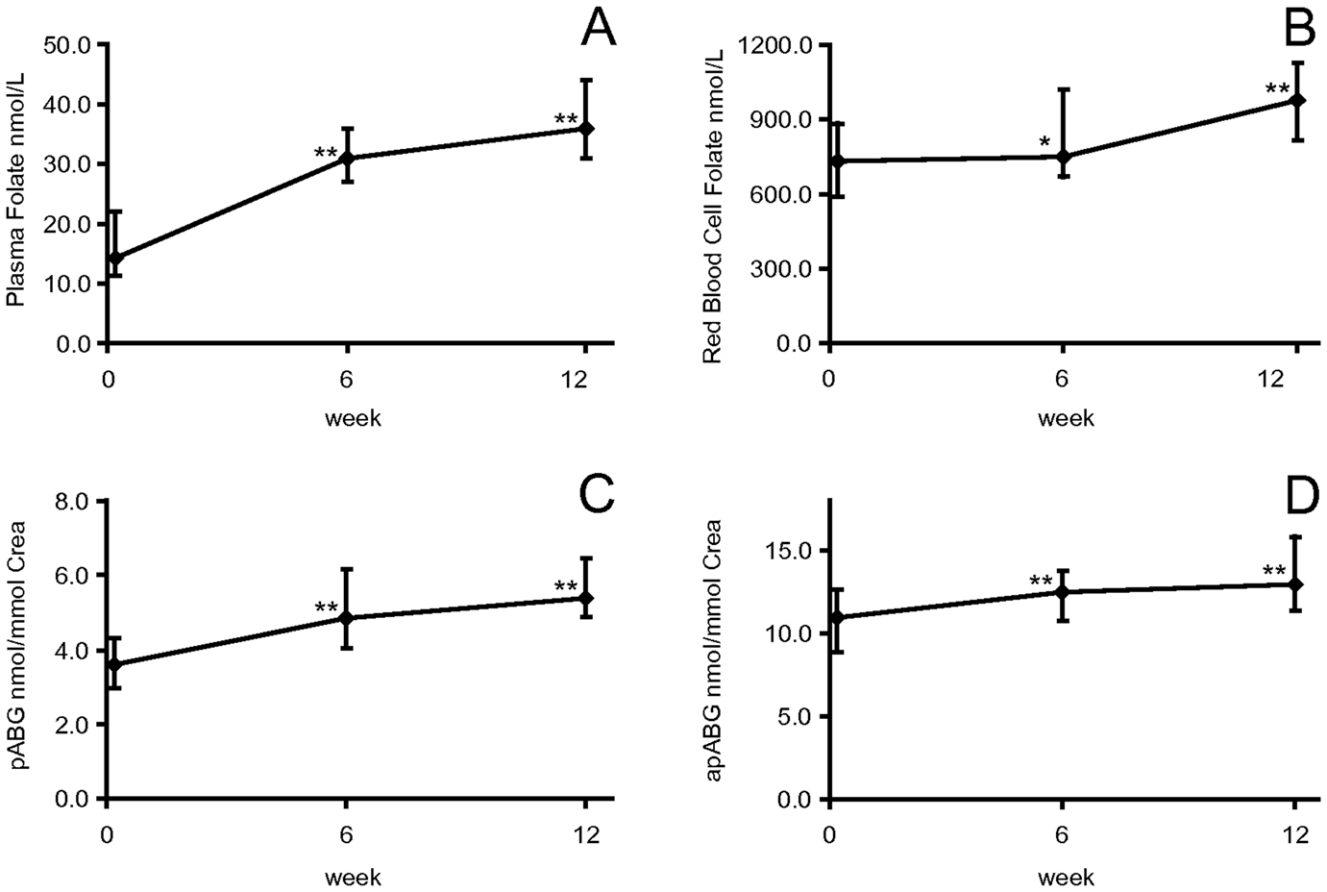
Time course of plasma, red blood cell folate, urinary pABG and apABG during folic acid supplementation. Time course of the median concentrations (error bars = inter quartile range (IQR)) of plasma folate (nmol/L) (A), red blood cell folate (nmol/L) (B), pABG (nmol/mmol creatinine) (C) and apABG (nmol/mmol creatinine) (D) during folic acid supplementation. Significant changes from baseline: *: p = 0.005 and **: p≤0.001 (Wilcoxon’s test).

Caudill et al. [41] found non-significantly higher urinary pABG excretion in non-pregnant women consuming 850 µg folate per day (mainly synthetic folic acid) than in women consuming 450 µg. We found highly significant increases of pABG (p≤0.001, Figure 4C) and apABG concentrations in spot urine (p≤0.001, Figure 4D) from baseline with habitual folate intake to week 6 and week 12 of supplementation in subjects supplementing 400 µg folic acid per day in addition. This is in agreement with data reported by Wolfe et al. [19], who only found significantly higher total pABG (sum of pABG and apABG) excretion after increasing daily folate intake for 7 weeks from 115 µg (depleted diet) to 400 µg per day.

Folic acid supplementation in the intervention study resulted in significant increases of RCF (p = 0.005, Figure 4B) and PF (p≤0.001, Figure 4A) from baseline to week 6. RCF increase during the first 6 weeks was significant, but lower than the increase between week 6 and week 12 (Figure 4B). Given this pattern of longitudinal change, a 12 week supplementation period with 400 µg folic acid seemed not sufficient to achieve a steady state of RCF concentrations as also reported by Houghton et al. [44].

PF can rapidly be influenced by dietary folate intake [45]. In our study PF increased mostly during the first 6 weeks of folic acid supplementation and less steeply from week 6 to week 12. Similarly, Hao et al. [46] observed a plateau for PF after 12 weeks of supplementation with 400 µg folic acid/d in young women.

### Folic Acid Supplementation Induced Changes of Correlations

The relationship between blood folates and urinary folate catabolite concentrations during folic acid supplementation in the intervention study was different from that observed at baseline (Figure 3D and 3F; Table 2). The correlation between RCF and pABG, still highly significant at baseline (r_s_ = 0.649, p<0.001), vanished during supplementation at week 6 (r_s_ = 0.074, p>0.05) and week 12 (r_s_ = 0.097, p>0.05). The relationship between RCF and apABG was also stronger at baseline (r_s_ = 0.543, p<0.001), but did not completely disappear during supplementation (week 6: r_s_ = 0.228, week 12: r_s_ = 0.343). The most stable association was observed between PF and pABG in spot urine, which was also highest at baseline (r_s_ = 0.676, p<0.001) and remained visible during folic acid treatment (week 6: r_s_ = 0.346, p>0.05, week 12: r_s_ = 0.574, p<0.05). In contrast, no significant correlation between PF and apABG was observed during intervention. Correspondingly, Wolfe et al. [19] reported significant correlations of serum folate and RCF with the sum of pABG and apABG excretion at study start and after 7 weeks of folate depletion, but did not find significant correlation after oral dosage of 400 µg/day for further 7 weeks.

**Table 2:** RCF, PF, urinary folate catabolites during folic acid supplementation (week 6, 12).

	week 6				week 12			
	Median (IQR) (n = 25)	Correlation with RCF (nmol/L)	Correlation with PF (nmol/L)	Significance level of changes frombaseline (p-value)	Median (IQR) (n = 25)	Correlation with RCF (nmol/L)	Correlation with PF (nmol/L)	Significance level of changes from baseline(p-value)
Folate, red blood cells (nmol/L)	748 (671; 1020)	1	0.520*	0.005	976 (816; 1130)	1	0.193	≤0.001
Folate, plasma (nmol/L)	31.0 (27.0; 36.0)	0.520*	1	≤0.001	36.0 (31.0; 44.0)	0.193	1	≤0.001
Homocysteine, plasma (µmol/L)	9.8 (9.5; 10.9)	0.264	−0.232	≤0.05	10.1 (9.40; 11.7)	0.309	−0.408*	≤0.005
pABG, urine (nmol/mmol creatinine)	4.84 (4.04; 6.17)	0.074	0.346	≤0.001	5.37 (4.88; 6.44)	0.097	0.574*	≤0.001
apABG urine (nmol/mmol creatinine)	12.5 (10.7; 13.7)	0.228	0.195	≤0.001	13.0 (11.3; 15.8)	0.343	0.221	≤0.001

Values are given as median and IQR in parentheses, relations between variables were analyzed by Spearman’s rank correlation. pABG: para-aminobenzoylglutamate, apABG: para-acetamidobenzoylglutamate, RCF: red blood cell folate, PF: plasma folate. Spearman’s rank correlation significance level:

* p<0.05. Significance of changes from baseline was calculated using Wilcoxon’s test and given as p-value.

In our short-term study, a 400 µg oral bolus of folic acid substantially affected urinary catabolite concentrations. Previously, Prinz-Langenohl et al. [16] had shown that intake of 400 µg folic increased PF for a prolonged time period. Although it has been suggested that pABG partially originates from nonspecific extracellular folic acid degradation in the circulation [38], concentration variations in plasma and urine may follow different time courses and PF and urinary pABG may be differently biased by recent folate intake, depending on the timing of the last folic acid intake. The correlation between PF and pABG may have been attenuated further by excluding urine samples with a ratio_p/ap_ above 0.7, since these samples exhibited both exceptionally high values for PF and pABG.

It has been shown in postmenopausal women that RCF has a delayed and attenuated response to periods of folate depletion and repletion when compared to PF [47], while urinary pABG and apABG excretion only showed non-significant trends to change in response to the controlled variations of folic acid intake [34], [47]. This agrees with the assumption of kinetically different folate pools derived from tracer studies [32]. Thus, higher short-term variations of PF and catabolite concentrations in urine during supplementation than at baseline and the existence of several folate pools with different kinetic behavior may well explain the absence of significant correlations between plasma and urine markers after 6 weeks of supplementation. After 12 weeks of folic acid intake the correlation between PF and urinary pABG was similar to the correlation at baseline, which confirms that both parameters reflect fast changing pools [20]. Furthermore, diurnal variations of PF and pABG might have become less pronounced after 6 more weeks on constantly elevated folic acid intake. On the other hand the quantitatively major folate catabolite apABG did not correlate with PF and RCF and one might infer from this that apABG is mainly derived from other, probably cellular, pools [48]. Furthermore, this finding would be consistent with the assumption that the red cell folate pool followed different kinetics than tissue folate pools, which has been shown in rats [36] but not studied in humans so far. It is of importance to note that different turnover of folate in red cells and major folate containing tissues such as liver, does not contradict with the well proven suitability of RCF to indicate body folate status in steady state.

In contrast to previous studies [19], [37], [41], we did not collect 24 hour urine during supplementation, because we assumed 24 hour excretion would not be more closely related to PF or RCF than fasted, second void morning urines, unless the subjects followed precisely a time schedule defining folic acid intake, start of collection period and blood sampling.

Thus, recent onset of bolus doses of folic acid in subjects with relatively low folate status seems to preclude reliable estimation of RCF from urinary folate catabolites, but the situation may be different after periods of more than 3 months of continuous supplement intake.

### Urinary Folate, Homocysteine and B- Vitamins

The very small urinary folate excretion per day in the baseline study was consistent with previous findings [32], [41]. Although folate excretion increased significantly with oral folic acid supplementation, the sum of urinary pABG and apABG exceeded urinary folate, as already shown by Gregory et al. [23], [35]. PF and RCF concentration were significantly correlated to urinary folate in spot urine and 24 hour urine at baseline (Table 1). After 12 week of folic acid supplementation, PF correlated with urinary folate in spot urine (r_s_ = 0.420, p<0.05). Compared to urinary folate catabolites, measuring urine folate for estimation of folate status has several important disadvantages: Urinary folates are much less stable than folate catabolites [49] and therefore determination in large-scale studies is likely to be severely biased by sampling conditions. Urinary folates are also much more influenced by immediate dietary intake of folates [50] warranting very strict control of sample timing. On the other hand, folate catabolites, namely apABG, are better correlated to mid-term folate storage pools (RBC folate, tissue folate [32], in contrast to urinary folate which follows short-term variations in plasma folate.

82.4% of all participants had homocysteine levels lower than 15 µmol/L, which is recommended for adults [6], [7]. Only one participant showed a homocysteine level above 30 µmol/L, which indicated hyperhomocysteinemia [6]. While significant negative correlation (r_s_ = −0.420; p<0.001) between PF and homocysteine was found, there was no significant correlation between RCF and homocysteine in plasma at baseline. As observed previously [51], homocysteine levels decreased significantly (p<0.05) during supplementation (Table 2). Wald et al. [51] reported lately that the higher a subject’s initial homocysteine level, the larger it’s decrease in response to folic acid supplementation. According to that we also detected slightly higher response to the 12 week daily folic acid intake in a subgroup with elevated homocysteine levels at baseline (homocysteine >15 µmol/L), while subjects with normal baseline values (<15 µmol/L) exhibited only minor changes of plasma homocysteine during intervention. As Lobo et al. [52] already observed in a group taking 400 µg folic acid per day, decrease of homocysteine levels directly after starting supplementation in our subjects was higher (p<0.05) than those between week 6 and 12 of daily folic acid intake (Table 2). But maintained supplementation up to 12 weeks furthermore increased the significance of chances (p<0.005) compared to baseline levels.

Measurements of pyridoxal phosphate, cobalamin and urinary methylmalonic acid provided no indication of vitamin B_6_ or B_12_ deficiency in any of the participants [29], [53]. Since no significant correlations between vitamins B_6_ and B_12_ and blood and urine based markers of folate status were detected, we assumed that these vitamins did not influence the relationship between folate status markers.

### Limitations of the Study

A major shortcoming of the current study, which only studied well-nourished Europeans (Germans), was that none of the subjects exhibited clearly in-adequate folate status, i.e. all individuals had RBC folate concentrations >400 nmol/L. Due to this limitation, we were not able to determine cut-of values of urinary folate catabolites indicating inadequate folate status.

Further investigations in populations with insufficient folate intake might provide information for determining such cut-off points.

The inclusion of healthy middle-aged men only, which somewhat limits generalizability of results, was guided by the intention of providing the most homogenous study population for systematically addressing baseline variation and intervention effects. Including women might have led to a more inhomogeneous group (due to folate-containing oral contraceptives [54], pregnancy or lactation [55]) and studying children seemed ethically inappropriate.

Using LC-MS/MS for analysis of folate catabolites might seem to limit availability of the proposed assay to specialized laboratories, but this technology stands out in delivering an unprecedented combination of sensitivity and selectivity with relatively low costs per sample. Furthermore, LC-MS/MS has become more and more widely available in recent years, since it has already been introduced into routine laboratories [56].

### Conclusions

Our results in non-folic acid supplemented subjects show that urinary pABG normalized to creatinine in spot urine is more closely related to PF than apABG whereas urinary concentrations of both folate catabolites showed comparable correlations with RCF. This suggests that pABG/creatinine in spot urine is suitable as a non-invasive biomarker for folate status. A fasting period of more than 12 hours before sampling of the second fasted morning spot urine is recommended to exclude overestimation of folate status due to recent folate intake. The property of folate catabolites to be stable at ambient temperature in urine samples for several days makes collection of urine samples an attractive choice for non-invasive field studies. In general, measuring folate catabolites instead of folates involves much less variation due to sampling and measurement errors since folate catabolites do not share the well-known chemical instability of folate species. The ratio of pABG to apABG in urine appears to be a suitable marker of sampling conditions compliance. This ratio is rapidly increased by oral intake of folates enabling identification and exclusion of biased urine samples not suitable for reliable estimation of folate status. Folic acid supplementation up to 12 weeks attenuates correlations between urinary and blood folate markers. Thus, only the fast adapting folate compartment PF can be probed non-invasively during supplementation by pABG determination in urine while RCF and apABG seem to reflect different slowly adapting pools.

Our results suggest further investigation of the relationship between folate status-related disease risks and pABG and apABG in spot urine of subjects with habitual folate intake, since it potentially may replace invasive blood sampling as well as error-prone 24 hour urine collection in medical diagnosis and clinical trials.

## Supporting Information

Document S1 **Study Protocol: “Determination of the suitability of urinary total p-aminobenzoyglutamate and formiminoglutamate as a markers for folate status”.**
(PDF)

Document S2 **Funding Sources.**
(PDF)

Document S3 **CONSORT Checklist.**
(PDF)
