# Supplementary material for: Folate Catabolites in Spot Urine as Non-Invasive Biomarkers of Folate Status during Habitual Intake and Folic Acid Supplementation
Source: PLoS One. 2013 Feb 14;8(2):e56194. doi: 10.1371/journal.pone.0056194 (PMC3572985; doi:10.1371/journal.pone.0056194)
Supplement: Document S1 — Study Protocol: “Determination of the suitability of urinary total p-aminobenzoyglutamate and formiminoglutamate as a markers for folate status”. (PDF) [file pone.0056194.s001.pdf]

Determination of the suitability of urinary total p-aminobenzoyglutamate and formiminoglutamate as a markers for folate status

[1]Hans Demmelmair, [1]Wolfgang Peissner, [1]Thea Weith, [2]Diego Moretti, [2]Petra Verhoef, [1]Astrid Rauh-Pfeiffer, [1]Berthold Koletzko

[1]Labor für Stoffwechsel und Ernährungsforschung, Dr. von Haunersches Kinderspital, Klinikum der Universität München, Germany,
[2]Unilever Food and Health Research Institute, Olivier van Northlaan 120 PO box 114, 3130 AC Vlaardingen

1. Summary 2

2.Investigators and financial support 3

3. Introduction 3

4. Objectives 6

5. Participating subjects 6

6. Study Procedure 7

7. Study supplement 11

8. Laboratory analyses: 11

9. Data management and biometrical evaluation 13

10. Ethical consideration, safety and insurance 14

11. References 15

1. Summary

Folate intake may be marginal in subgroups of the western population, and a noninvasive method for the screening of folate status would allow for easier and less cumbersome assessment, than the identification via the established markers serum or red blood cell folate concentration. Such a method could be applied in epidemiological studies and in large clinical trials including children.

Markers, which seem suitable for this purpose are urinary excretion of formiminoglutamate (FIGLU) and para-aminobenzoylglutamate (p-ABG), which are an intermediate of folate dependent conversion of histidine to glutamate or a catabolite of folate, respectively. In case of folate deficiency urinary excretion of FIGLU is increased after a histidine load and clinical trials have shown increased total p-ABG excretion after increased folic acid intake. Thus we plan to test the suitability of these markers by analyzing red blood cell and serum folate concentration as well as 24-h urinary excretion of FIGLU and p-ABG in 50 healthy, adult men. The participants will deliver a spot urine sample and a blood sample at their first study visit, while at the second visit (21 days later) they will provide the collected urine of the last 24 hours, a further spot urine sample and a second blood sample. This enables testing of short time variation of circulating markers. Furthermore, this enables a comparison of the results obtained by direct measurement of 24-h urinary excretion with estimation of 24-h excretion from a single sample with adjustment via urinary creatinine concentration. These measurements are used to evaluate the applicability of urinary FIGLU and p-ABG as indicators of folate status for screening purposes.

In the supplementation part of the study from those participants in the lowest quartile of RBC folate level and from those in the highest quartile of RBC folate 10 subjects will be selected each, for participation in a folic acid supplementation trial. These subjects will consume daily a 400 µg folate supplement for 12 weeks. Their urinary and blood folate markers will be analysed after 6 weeks and 12 weeks to evaluate the suitability of FIGLU and p-ABG excretion as indicators of folate level in supplementation studies. The relationship between circulating and urinary markers will be statistically evaluated by analysis of correlation and by receiver operator characteristics analysis.

If suitability of these new markers could be demonstrated, this would provide an additional tool for folate screening and for use in clinical trials.

.

2. Investigators and financial support

Principal investigator:

Professor Berthold Koletzko

Div. Metabolic Diseases and Nutritional Medicine

Dr. von Hauner Children’s Hospital, Ludwig-Maximilians-University of Munich

Lindwurmstr. 4, D-80337 Muenchen

Tel: +49 (0)89 5160 2826, Fax: +49 (0)89 5160 3336

E-mail: claudia.wellbrock@med.uni-muenchen.de .

Finacial support:

- European Union – DG Research via FP7 project “NUTRIMENTHE“
  (Grant agreement nº: 212652)

3. Introduction

The importance of folate mediated one-carbon metabolism in metabolic and cellular processes has long been recognized and epidemiological, genetic and biochemical studies have revealed that folate status is related to the incidence of certain cancers, birth defects and cardiovascular disease (Stover, 2004). Folate occurs in the cells as several structurally related derivatives comprised of 2-amino-4-hydroxypteridine linked to p-aminobenzoylglutamate. Reduced tetrahydrofolates serve as cofactors that deliver one-carbon units at different oxidation levels. Reduced tetrahydrofolate may have a one-carbon substituent, such as a methyl-, formyl- or formimino- group at N5 or a methylene or methenyl bridge between N5 and N10. These substituents form a pool of activated one carbon units. They are transferred to other molecules in a variety of biochemical reactions. While in serum folate has a monoglutamyl residue, intracellularly folate usually occurs with 5–8 γ-linked glutamyl residues. The polyglutamate moiety seems important for proper function of the coenzyme. Most of the intracellular folate by far is protein bound (Matherly et al, 1990). In rats it has been demonstrated that tight folate binding proteins are highly or exclusively expressed in the liver, thus liver may contain up to one half of total body folate (Gregory, III et al, 1998). As non protein bound intracellular folate is negligible, this also indicates that intracellular folate is limited by folate-binding capacity and independent of excessive exogenous folate supply (Suh et al, 2001).

Folate is important for the transfer of one-carbon units, as required for the de novo synthesis of purines and thymidine and for the remethylation of homocysteine to yield methionine. Further biochemical reactions, which require folate as a cofactor are conversion of serine to glycine and histidine breakdown, yielding as an intermediate γ-

formiminoglutamic acid (FIGLU), which needs folic acid as a formyl acceptor for conversion to glutamic acid.

As mammals can not synthesize folate de novo, a dietary supply is required. Naturally occurring folate has a reduced pteridine ring and a polyglutamate residue, which has to be hydrolysed intestinally to monoglutamate before absorption, while synthetic folate is provided with a fully oxidised pteridine ring and only one glutamate residue. After reduction to tetrahydrofolate it is equivalent in its biological function to natural follate.

Folate deficiency disrupts folate requiring pathways, such as homocysteine remethylation and thymidylate synthesis, and leads to identifiable pathologies. Thus, plasma homocysteine level is a sensitive biomarker for folate status, if availability of vitamins B2 (riboflavin), B6 (pyridoxine) and B12 (cobalamin) is adequate (Brosnan, 2004). Impaired conversion of homocysteine to methionine leads to accumulation of S-adenosylhomocysteine, which inhibits S-adenosylmethionine dependant reactions, including DNA methylation. The relationship between folate status and neural tube defects is well established, with folic acid supplementation decreasing the incidence significantly (Tamura et al, 2006).

Direct biochemical indicators of folate status are serum and red blood cell (RBC) folate levels. Serum folate represents circulating folate, which can change quickly and is directly influenced by diet, whereas RBC folate level is assumed to represent folate stores (Hoey et al, 2007). RBC folate is a good indicator of long term status, because cellular folate stores accumulated during erytropoesis are retained throughout the lifespan of the erythrocyte. It has been demonstrated in rats that RBC and hepatocyte folate levels strongly correlate (Picciano et al, 1995). In humans a clear relationship between RBC folate and the incidence of neural tube defects could be shown (Daly et al, 1995).

Beyond these established markers, it would be desirable to obtain a reliable estimate of the folate status via non invasive markers, which can easily be collected and applied in a large number of subjects including children. Folate intake may be marginal in subgroups of the western population, and a noninvasive method for the screening of folate status would allow for easier and less cumbersome assessment. Furthermore, such a screening tool would allow to assess, if people are exposed to high doses of folic acid, which has been raised as a concern in elderly, in countries with folic acid fortification. Buccal cell folate has been considered suitable, but correlation with RBC folate has been found to be poor and buccal mucosa folate did not show any effect of 12 weeks of supplementation with 1.2 mg folic acid per day (Basten et al, 2004). As an alternative, urine can be sampled noninvasively and concentrations of folate and homocysteine can be determined. Urinary homocysteine concentrations seem not to be related to plasma total homocysteine concentrations (personal communication, P. Pencharz, Paediatrics and Nutritional

Sciences, University of Toronto, Canada; and P.M. Ueland, Department of Pharmacology, University of Bergen, Norway). 24-h urinary excretion of folate was reported to decline significantly in Mexican American men within 6 weeks after onset of a controlled diet, which provided 418 µg/d of dietary folate equivalents (Solis et al, 2008). At the same time significant decreases of serum and RBC folate were observed. Although this suggests applicability of urinary folate excretion as an indicator of folate status, there is large interpersonal variation in the amount excreted and excretion might depend on methyltetrahydrofolatereductase genotype (Yang et al, 2005; Guinotte et al, 2003). This seems to limit the predictive value of folate excretion for folate status. Urinary excretion of FIGLU is known to be elevated after a histidine load, in case of folate deficiency (Cooperman et al, 2002). Although in a previous study no significant correlation of FIGLU excretion, with other markers of folate status could be demonstrated (Pietrzik et al, 1980), improved analytical techniques (LC-MS/MS) warrant a reconsideration. However, the most promising biomarker of folate status in urine might be folate catabolites (Bailey et al, 1999). Although not polyglutamated folate can efflux from the cell either directly after entering the cell or after hydrolysis by γ-glutamylhydrolase, non reversible folate catabolism proceeds via non enzymatic or potentially enzyme catalysed cleavage of the C9-N10 bond of dihydrofolate, yielding a peterin aldehyde and p-aminobenzoylglutamate (Suh et al, 2001). While the pterin residue is retained in the liver and only slowly released, the export of p-aminobenzoylglutamate (p-ABG) out of the cells is facilitated by removal of the polyglutamate and the majority of p-ABG is acetylated at the amino group catalysed by arylamine N-acetyltransferase. Acetylated p-ABG (Ap-ABG) does not accumulate in cells and is rapidly excreted in urine (Murphy et al, 1976). In rats Ap-ABG concentration in urine is considered a reliable marker of folate status and turnover (Wang et al, 1994). Results in humans are not fully supporting the hypothesis that urinary total p-ABG is a good indicator of folate status and/or intake, because catabolite excretion might reflect changed intake only after some delay as total p-ABG (Ap-ABG and p-ABG) originates from slow turnover pools (Gregory, III et al, 2000). Thus, effects of short term interventions might not be detected by the analysis of urinary p-ABG, but a correlation with a long term marker such as RBC folate, can be assumed. Furthermore, tracer experiments with deuterated folic acid demonstrated that turnover of folate pools in humans increases with increasing intake, thus an increased catabolite excretion can be expected (Gregory, III et al, 1998). In a study in postmenopausal women, involving 7 week periods of folate depletion (intake 120 µg/d) and repletion (intake 200 µg/d or 400 µg/d) significant correlations between serum folate, RBC folate and urinary excretion of catabolites were observed (Wolfe et al, 2003). These findings were obtained with 24h urine collections, thus it will be tested whether FIGLU or total p-ABG concentrations in spot urine samples can be extrapolated to 24 h excretion via urinary creatinine concentration (Bingham et al, 1988). This is of

importance as in large clinical trials or in population screening only collection of spot samples is feasible.

So far wider application of FIGLU and p-ABG as markers in clincial studies was limited by demanding analytical procedure (McPartlin et al, 1992), but with the availability of a LC-MS/MS based methods (Sokoro et al, 2006; Bishop et al, 2007; Hannisdal et al, 2008), which enable high sample throughput, application of urinary FIGLU and p-ABG as a non-invasive markers of folate status has become possible and corresponding validation studies are warranted.

4. Objectives

The major objective of the proposed study is to demonstrate the suitability of new markers for folate status in humans, which avoid blood sampling, as required for the established marker RBC folate concentration, but can be obtained from spot urine samples.

Specific hypotheses to be tested:

Correlation study:

a) The 24 h urinary excretion of total p-ABG, as extrapolated via urinary creatinine concentration from the total p-ABG concentration in a spot sample is significantly correlated to RBC and plasma folate levels in the studied subjects
b) The 24 h urinary excretion of FIGLU, as extrapolated via urinary creatinine concentration form the FIGLU concentration in a spot sample is significantly correlated to RBC and plasma folate levels in the studied subjects

Intervention study:

a) changes in folate status (RBC folate) after supplementary intake of folate result in increased urinary excretion of total p-ABG and FIGLU in subjects identified with a low folate status and in subjects identified with a high folate status among the study participants of the correlation study

5. Participating subjects

5.1. Subject number

The sample size estimation is based the correlation study. It is assumed that a correlation of at least 0.35 would be sufficient to use urinary total p-ABG or FIGLU as markers for

folate status. To detect such a correlation as statistically significant with a power 80% and α of 0.05 samples from 49 subjects have to be analyzed (Uitenbroek, Daan G, Binomial. SISA. 1997. http://www.quantitativeskills.com/sisa/distributions/binomial.htm. (1 Jan. 2004). Thus, 50 subjects will be enrolled into the correlation study and from these participants for the intervention study will be selected.

5.2 Selection criteria and recruitment

The subjects to participate will be contacted by advertisement on the campus of the University of Munich, by announcing the study on the website of the university hospital and if needed by newspaper advertisement. For enrollment subjects have to fulfill the following inclusion criteria:

- age: 18 – 45 years
- apparently healthy
- normal results on haematologic pattern and blood chemistry tests (according to university hospital reference values)
- plasma vitamin B12 > 160 pmol/l
- no diagnosis of intestinal, renal or thyroid disease (self reported)
- only male subjects will be enrolled but regular consumption of vitamin supplements (including folate) is not an exclusion criterium, as we aim to cover a wide range of folate concentrations.

On the other hand, conditions, which are expected to influence folate catabolism are exclusion criteria:

- recent (3 months) treatment with medication, assumed to interfere with folate status
- abuse of alcohol or drugs

6. Study Procedure

The study consists of two consecutive parts (Fig. 1). During the initial correlation study the relationship between established markers of folate status and the newly proposed markers (urinary FIGLU and urinary total p-ABG) is investigated. From the subjects participating in the correlation study, persons with low and high folate status will be selected for participation in the intervention study.

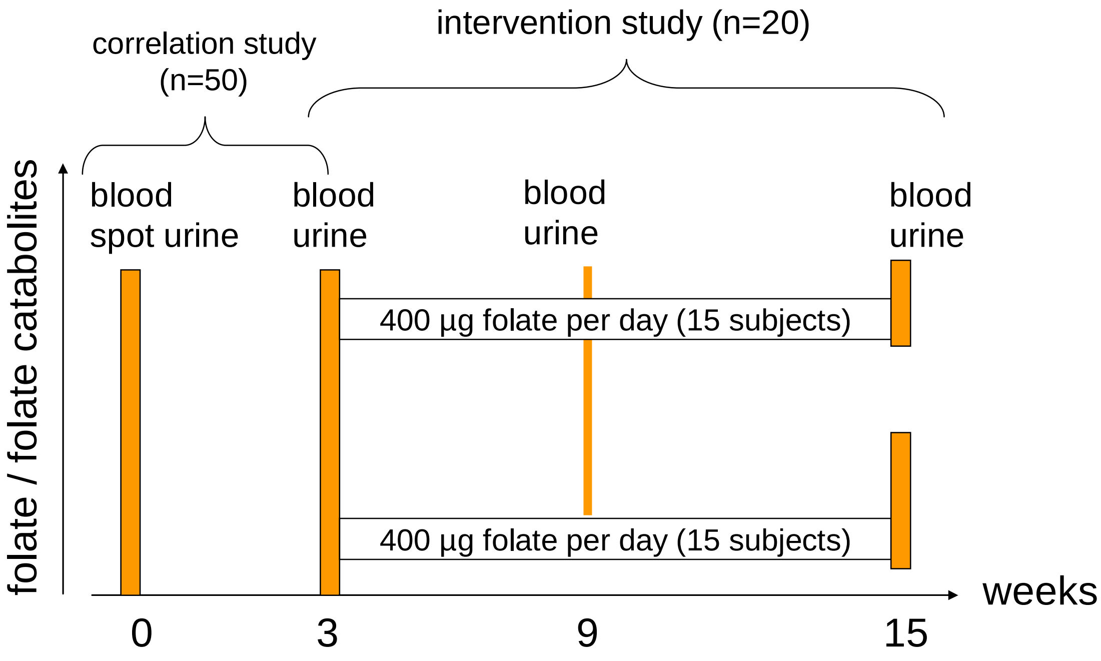

Figure 1: Overview about the study procedure including the correlation study and the intervention study, which involves only selected subgroups of the participants of the correlation study

Potential subjects will contact the study center via telephone and during the telephone conversation eligibility criteria will be preliminarily checked and questions of the subjects in respect to the study procedure and associated burden will be answered. If criteria seem fulfilled and the person intends to participate an appointment will be made for obtaining informed consent and if obtained blood and urine will be sampled. These appointments will be before 9:00 in the morning (to obtain fasted blood samples) in the Dr. von Hauner Children Hospital. At this initial appointment blood and spot urine will be collected. Furthermore the subjects are provided with a questionnaire on their dietary habits, with a focus on folate containing food stuffs and vitamin supplements. The questionnaire will also cover any medication taken and principle parameters of a physical examination and anthropometry will be recorded. The completed questionnaire will be brought back at the second blood sampling appointment, which will be scheduled 3 weeks later also early in the morning. The subjects will also be given a suitable container for a full 24 urine collection. Starting in the morning on the day before the second blood sampling subjects start a complete urine collection and hand over the urine to the study personal at the second blood sampling appointment, which follows the same procedure as the first appontment. The questionnaire will be briefly discussed with the subjects to clarify any obvious ambiguities.

6.1 Correlation study

Detailed planning:

1. Interested subjects will call the study center for preliminary discussion of the study and eligibility criteria; if the subject seems eligible and intends to participate details for the first appointment will be agreed and instructions will be given
2. First appointment (before 9.00 am after a 12 hour fasting period):
- written informed consent form will be obtained (covering correlation study and intervention study)
- physical examination and clinical questionnaire will be completed
- collection of cheek cells with a sterile swab (09-511-5006/2, nerbe plus GmbH) according to clinical routine and storage of the sample at -20ºC
- fasted blood will be collected by venipuncture (serum: S-Monvette 05.1104, 4.5 ml, EDTA blood: S-Monovette, 4.9 ml)
  EDTA- blood for hematology and analyses from whole blood will be taken away and the remaining blood samples will be centrifuged (4ºC, 1400g, 5 min) to obtain serum and plasma, which will be transferred to the respective laboratories or frozen at -80ºC for storage and later analysis
- spot urine will be collected in a brown 100 ml beaker (Sarstedt: 75.562.011) and 3 aliquots (10 ml each, Sarstedt 10.252.030) will be frozen for later analysis at -20ºC
- questionnaire on dietary habits (focus on folate intake) will be handed over to the subjects for completion and returning at the second appointment
- container (2.0 l, Sarstedt 77.580, containing 5 g ascorbate to prevent folate breakdown) and instructions for 24h urine collection will be handed over to the subject
3. Second appointment (before 9.00 am, after a 12 h fasting period, 3 weeks after first appointment):
- dietary questionnaire will be returned and checked for plausibility
- 24h urine container returned, the volume of urine will be recorded and after thorough mixing 3 aliquots (10 ml each, Sarstedt 10.252.030) will be frozen for later analysis at -20ºC
- additionally spot urine will be collected in a brown 100 ml beaker (Sarstedt: 75.562.011) and 3 aliquots (10 ml each, Sarstedt 10.252.030) will be frozen for later analysis at -20ºC
- fasted blood will be collected by venipuncture (serum: S-Monvette 05.1104, 4.5 ml, EDTA blood: S-Monovette, 4.9 ml)
  EDTA- blood for hematology and for analyses from whole blood will be taken away and

the remaining blood samples will be centrifuged (4ºC, 1400g, 5 min) to obtain serum and plasma, which will be transported to the respective laboratories or frozen at -80ºC for storage and later analysis

- subjects qualifying for the intervention study will be enrolled (see 6.2.)

6.2 Intervention study

The part of the study described so far (correlation study) relates to the evaluation of the principal relationship between urinary excretion of total p-ABG and FIGLU and blood markers of folate status, which is needed for the evaluation of the suitability of these markers as screening tools and as proxies for folate status. The second part (intervention study) shall clarify, whether total p-ABG and FIGLU excretion are suitable for the determination of changes in folate status after increased intake of folic acid starting from relatively high or low levels.

From those subjects, who do not already take supplements, 15 persons each from the highest and lowest quartile in respect to RBC folate status (based on RBC folate concentration at the first appointment) will be asked to participate in a 12 week supplementation study. The participating subjects will be given a folate supplement, providing 400 µg of folic acid per day. For the evaluation of the time course of the blood and urine markers of folate status after 6 weeks and after 12 weeks blood and urine samples will be obtained. During the intervention period subjects will be contacted by telephone regularly to discuss questions and to enhance adherence to the protocol.

1. Second appointment (continued):

- subjects (n=20, 10 each for the highest and lowest quartile of RBC folate status) who participate in the intervention study will be given a pack with 50 tablets (400 µg folate/tablet) of a commercially available folate supplement
- a brief questionnaire to note any unusual events or complaints will be provided
- container (2.0 l, Sarstedt 77.580, containing 5 g ascorbate to prevent folate breakdown) and instructions for 24h urine collection will be handed over to the subject (if collection of spot samples is found useful in the correlation study spot samples will be collected instead of the 24h urine)
- a third appointment about 6 weeks later will be scheduled

2. Third appointment (intervention study participants only, before 9.am after a 12 h fasting period, 6 weeks after second appointment)

- questionnaire on events will be discussed

- the urine container will be returned, the volume of urine will be recorded and after thorough mixing 3 aliquots (10 ml each, Sarstedt 10.252.030) will be frozen for later analysis at -20ºC
- fasted blood will be collected by venipuncture (serum: S-Monvette 05.1104, 4.5 ml, EDTA blood: S-Monovette, 4.9 ml)
  EDTA- blood for hematology and analyses from whole blood will be taken away and the remaining blood samples will be centrifuged (4ºC, 1400g, 5 min) to obtain serum and plasma, which will be transported to the respective laboratories or frozen at -80ºC for storage and later analysis
- the left over tablets will be counted and the participants will be given a second pack with 50 tablets (400 µg folate/tablet) of a commercially available folate supplement
- a fourth appointment about 6 weeks later will be scheduled

3. Fourth (final) appointment (intervention study participants only, before 9.am after a 12 h fasting period, 6 weeks after third appointment)
- questionnaire on events will be returned and discussed
- the urine container will be returned, the volume of urine will be recorded and after through mixing 3 aliquots (10 ml each, Sarstedt 10.252.030) will be frozen for later analysis at -20ºC
- fasted blood sample will be collected by venipuncture (serum: S-Monvette 05.1104, 4.5 ml, EDTA blood: S-Monovette, 4.9 ml)
  EDTA- blood for hematology and analyses from whole blood will be taken away and the remaining blood samples will be centrifuged (4ºC, 1400g, 5 min) to obtain serum and plasma, which will be transported to the respective laboratories or frozen at -80ºC for storage until later analysis
- not consumed supplement tablets will be returned and counted

7. Study supplement

The study supplement will be folate containing tablets approved as non prescription vitamin supplement, which provide 400 µg folate per tablet (Folverlan® 0.4mg, Verla-Pharm Arzneimittel, 82234 Tutzing). The subjects will consume one tablet per day.

8. Laboratory analyses:

Analyses will be conducted in the Labor für Stoffwechsel und Ernährungsforschung at the Dr. von Hauner Children Hospital of the University of Munich or in the central laboratories of the Dr von Hauner Children’s Hospital or the University Hospital, respectively.

a) full blood count and blood chemistry (ALAT, ASAT) will be determined at the central laboratory of the Children's Hospital according to established routine procedures

b) serum vitamin B12 concentration will be determined in the central laboratory of the university hospital by competitive protein binding and electro-chemiluminescence applying a commercial assay (Roche Elecsys; Mannheim, Germany)

c) plasma vitamin B6 (pyridoxine) will be determined in the central laboratory of the university hospital by HPLC applying a commercially available assay (Chromsystems, Munich, Germany), 300 µl EDTA plasma are required for analysis (transport at 4ºC with protection against light)

d) serum folate will be determined in the central laboratory of the university hospital by competitive protein binding and electro-chemiluminescence applying a commercial assay (Immolite 2000, Siemens ; Munich, Germany) .

e) RBC folate will be determined in the central laboratory of the university hospital by competitive protein binding and electro-chemiluminescence after lysis and dilution of the full blood applying a commercial assay (Immolite 2000, Siemens ; Munich, Germany)

f) plasma homocysteine will be determined in the central laboratory of the university hospital from EDTA blood using a competitive immunoassay employing direct chemiluminescence (ADVIA Centaur Homocystein Assay, Siemens Healthcare, Deerfiled, USA). The samples will be frozen at -80º and transported and analysed in batches.

g) urinary creatinine will be determined at the central laboratory of the children's hospital according to established routine procedures

h) urinary FIGLU analysis will be done in the Labor für Stoffwechsel und Ernährungsforschung by LC-MS/MS based on the procedure described by (Bishop et al, 2007)

i) urinary total p-ABG (Ap-ABG and p-ABG) will be determined in the Labor für Stoffwechsel und Ernährung using a LC-MS/MS method modified from Sokoro et al (Sokoro et al, 2006)

j) urinary methylmalonic acid will be analysed in the Labor für Stoffwechsel und Ernährungsforschung by LC-MS/MS

9. Data management and biometrical evaluation

All clinical data will be recorded in specifically prepared forms to be incorporated later into an electronic database. In the database they will be combined with the results of the laboratory analyses.

For all quantitative parameters determined a test for normal distribution will be performed according to Kolmogorov-Smirnov. If normal distribution can be assumed, mean and standard deviation will be given for these parameters. For non normally distributed data median and interquartile range will be provided and data will transformed appropriately prior to statistical analysis.

The repeated basal determination of blood markers of folate status is performed, as it is reasoned that both parameters undergo not only random variation, but there might be trends over time, after changes of dietary habits or physiological status. The effect of these trends should be attenuated by application of the average values of two measurements taken at different time points. This should become obvious from a better correlation of the averaged values compared to the individual values. A similar reasoning is behind the complete 24h urine collection, if there are short term variations in urinary concentration of total p-ABG or FIGLU extrapolation to total volume of excretion during 24 hours via urinary creatinine concentration is not adequate. If this is the case, there should be a closer relationship between the measured 24 h excretion of p-ABG and FIGLU with RBC folate than between the 24 h excretion estimated from the spot urine sample and RBC folate.

The major focus of statistical evaluation is a correlation and receiver operator characteristics (ROC) (Streiner et al, 2007) analysis between the determined circulating folate status markers and the urinary markers of folate status. Correlation analysis will be preformed according to Pearson or Spearman. For ROC analysis classification into the lowest quartile as assessed with RBC folate will be set as “Gold Standard”. Corresponding specificity, sensitivity, positive predictive value and negative predictive value will be determined for urinary total p-ABG and FIGLU excretion (measured and estimated 24 h excretion). Parameters will be compared in respect to the area under the ROC curve.

The obtained coefficients are the primary criterion for the applicability of the latter parameters as indicators of the subjects folate status. For a more detailed analysis of the data multiple regression analysis with RBC folate as the independent parameter and urinary excretion of total p-ABG or FIGLU as dependent variable including correction for other potentially confounding factors (e.g. vitamin B12, vitamin B6, age) will be performed. For these detailed analyses the support of an experienced biostatistician will be sought.

For evaluation of the time course of blood and urine markers of folate status repeated measures analysis of variance will be performed, with group allocation (high or low folate status according to RBC folate) as an independent factor. Furthermore, correlation analysis as described for the basal values will be performed, to investigate the suitability of urinary folate markers under conditions of a defined recent change of folate intake.

10. Ethical consideration, safety and insurance

The major aim of the study is to evaluate a non invasive measure of folate status, which is applicable in children and large epidemiological trials. Although theoretical considerations and study results point towards the usability of urinary total p-ABG and FIGLU excretion as folate status markers, its application in subjects on habitual and variable diets has to be tested . Furthermore, it is important to establish the indicative value of spot urine samples, as 24h urine collections seem not possible in screening studies. According to the best of our knowledge, it has not been tried before to establish the correlation of urinary markers and blood folate markers in healthy adult males on habitual diet and free choice to consume folic acid supplements. Establishment of the correlation between the established marker and a newly proposed marker, as well as the establishment of cut-off values for the new marker, are mandatory before the application of the new marker as a screening tool in a study, which aims to identify subjects with marginal folate status. The follow up of the changes of these markers after introduction of folate supplementation is a possibility of external validation of the marker, might establish upper limits of folate status for its applicability and tests its suitability as analytical parameter in clinical studies. Thus, the proposed protocol has the potential to collect valuable scientific data and achieve progress in nutritional science.

On the other hand risk and burden for the participating volunteers are limited. The risk associated with blood sampling is marginal and urine collection is cumbersome and inconvenient, but without risk. As vitamin B12 status of the subjects is continuously monitored and deficient subjects are excluded the additional intake of 400 µg folic acid daily can be considered as safe.

In conclusion, we predict that the data obtained from this study valuably contribute to the improvement of nutritional science without significant risk for the subjects and thus the study seems ethically well justified. The study will be conducted according to the established regulations including the registration of clinical trials, the coverage by an appropriate insurance and reporting of the results in a scientific publication.

11. References

(1) Stover PJ. Physiology of folate and vitamin B12 in health and disease. Nutr Rev 2004 Jun;62(6 Pt 2):S3-12.

(2) Matherly LH, Czajkowski CA, Muench SP, Psiakis JT. Role for cytosolic folate-binding proteins in the compartmentation of endogenous tetrahydrofolates and the 5-formyl tetrahydrofolate-mediated enhancement of 5-fluoro-2'-deoxyuridine antitumor activity in vitro. Cancer Res 1990 Jun 1;50(11):3262-9.

(3) Gregory JF, III, Williamson J, Liao JF, Bailey LB, Toth JP. Kinetic model of folate metabolism in nonpregnant women consuming [2H2]folic acid: isotopic labeling of urinary folate and the catabolite para-acetamidobenzoylglutamate indicates slow, intake-dependent, turnover of folate pools. J Nutr 1998 Nov;128(11):1896-906.

(4) Suh JR, Herbig AK, Stover PJ. New perspectives on folate catabolism. Annu Rev Nutr 2001;21:255-82.

(5) Brosnan JT. Homocysteine and cardiovascular disease: interactions between nutrition, genetics and lifestyle. Can J Appl Physiol 2004 Dec;29(6):773-80.

(6) Tamura T, Picciano MF. Folate and human reproduction. Am J Clin Nutr 2006 May;83(5):993-1016.

(7) Hoey L, McNulty H, Askin N, Dunne A, Ward M, Pentieva K, et al. Effect of a voluntary food fortification policy on folate, related B vitamin status, and homocysteine in healthy adults. Am J Clin Nutr 2007 Nov;86(5):1405-13.

(8) Picciano MF, Stokstad ELR, Gregory JF, III. Folic acid metabolism in health and disease. New York: Wiley-Liss; 1995.

(9) Daly LE, Kirke PN, Molloy AM, Weir DG, Scott JM. Folate levels and neural tube defects. JAMA 1995;274:1698-702.

(10) Basten GP, Hill MH, Duthie SJ, Powers HJ. Effect of folic Acid supplementation on the folate status of buccal mucosa and lymphocytes. Cancer Epidemiol Biomarkers Prev 2004 Jul;13(7):1244-9.

(11) Solis C, Veenema K, Ivanov AA, Tran S, Li R, Wang W, et al. Folate intake at RDA levels is inadequate for Mexican American men with the methylenetetrahydrofolate reductase 677TT genotype. J Nutr 2008 Jan;138(1):67-72.

(12) Yang TL, Hung J, Caudill MA, Urrutia TF, Alamilla A, Perry CA, et al. A long-term controlled folate feeding study in young women supports the validity of the 1.7 multiplier in the dietary folate equivalency equation. J Nutr 2005 May;135(5):1139-45.

(13) Guinotte CL, Burns MG, Axume JA, Hata H, Urrutia TF, Alamilla A, et al. Methylenetetrahydrofolate reductase 677C-->T variant modulates folate status response to controlled folate intakes in young women. J Nutr 2003 May;133(5):1272-80.

(14) Cooperman JM, Lopez R. The role of histidine in the anemia of folate deficiency. Exp Biol Med (Maywood) 2002 Dec;227(11):998-1000.

(15) Pietrzik K, Urban G, Hotzel D. [Biochemical and hematological parameters for the estimation of folate status in man. 3. Importance of formiminoglutamic acid excretion in the urine in comparison to other measurements]. Int J Vitam Nutr Res 1980;50(3):261-6.

(16) Bailey LB, Gregory JF, III. Folate metabolism and requirements. J Nutr 1999 Apr;129(4):779-82.

(17) Murphy M, Keating M, Boyle P, Weir DG, Scott JM. The elucidation of the mechanism of folate catabolism in the rat. Biochem Biophys Res Commun 1976 Aug 23;71(4):1017-24.

(18) Wang C, Song S, Bailey LB, Gregory JF, III. Relationship between urinary excretion of p-aminobenzoylglutamate and folate status of growing rats. Nutr Res 1994;14:875-84.

(19) Gregory JF, III, Swendseid ME, Jacob RA. Urinary excretion of folate catabolites responds to changes in folate intake more slowly than plasma folate and homocysteine concentrations and lymphocyte DNA methylation in postmenopausal women. J Nutr 2000 Dec;130(12):2949-52.

(20) Wolfe JM, Bailey LB, Herrlinger-Garcia K, Theriaque DW, Gregory JF, III, Kauwell GP. Folate catabolite excretion is responsive to changes in dietary folate intake in elderly women. Am J Clin Nutr 2003 Apr;77(4):919-23.

(21) Bingham SA, Williams R, Cole TJ, Price CP, Cummings JH. Reference values for analytes of 24-h urine collections known to be complete. Ann Clin Biochem 1988 Nov;25 (Pt 6):610-9.

(22) McPartlin J, Courtney G, McNulty H, Weir D, Scott J. The quantitative analysis of endogenous folate catabolites in human urine. Anal Biochem 1992 Nov 1;206(2):256-61.

(23) Sokoro AA, Etter ML, Lepage J, Weist B, Eichhorst J, Lehotay DC. Simple method for the quantitative analysis of endogenous folate catabolites p-aminobenzoylglutamate (pABG) and its acetamido (apABG) derivative in human serum and urine by liquid chromatography-tandem mass spectrometry. J Chromatogr B Analyt Technol Biomed Life Sci 2006 Feb 17;832(1):9-16.

(24) Bishop MJ, Crow BS, Kovalcik KD, George J, Bralley JA. Quantification of urinary zwitterionic organic acids using weak-anion exchange chromatography with tandem MS detection. J Chromatogr B Analyt Technol Biomed Life Sci 2007 Apr 1;848(2):303-10.

(25) Hannisdal R, Svardal A, Ueland PM. Automation and Analytical Techniques: Measurement of Folate in Fresh and Archival Serum Samples as p-Aminobenzoylglutamate Equivalents. Clin Chem 2008 Feb 15.

(26) Streiner DL, Cairney J. What's under the ROC? An introduction to receiver operating characteristics curves. Can J Psychiatry 2007 Feb;52(2):121-8.
