# Supplementary material for: Folate Catabolites in Spot Urine as Non-Invasive Biomarkers of Folate Status during Habitual Intake and Folic Acid Supplementation
Source: PLoS One. 2013 Feb 14;8(2):e56194. doi: 10.1371/journal.pone.0056194 (PMC3572985; doi:10.1371/journal.pone.0056194)
Supplement: Document S2 — Funding Sources. (PDF) [file pone.0056194.s002.pdf]

This work was supported financially by the “Kompetenznetz Adipositas” (“Competence Network for Adiposity”) funded by the Federal Ministry of Education and Research (FKZ: 01GI1126). Further support by the Commission of the European Communities, within the 7th Framework Programme, NUTRIMENTHE, FP7-212652 EarlyNutrition, FP7-KBBE-2011-05 and by the Munich Center of Health Sciences (McHealth) is gratefully acknowledged. This manuscript does not necessarily reflect the views of the Commission and in no way anticipates the future policy in this area. Berthold Koletzko has a received a Freedom to Discover Award of the Bristol-Myers Squibb Foundation, New York, NY, USA.
